# Translation and Validation of the Italian Version of the Team-Based Learning Student Assessment Instrument (TBL-SAI) in Nursing Students

**DOI:** 10.3390/nursrep15010026

**Published:** 2025-01-17

**Authors:** Valeria Vannini, Rosario Caruso, Sara Alberti, Sergio Rovesti, Paola Ferri

**Affiliations:** 1 Clinical and Experimental Medicine PhD Program, Department of Biomedical, Metabolic and Neural Sciences, University of Modena and Reggio Emilia, 287 Giuseppe Campi Street, 41125 Modena, Italy; sara.alberti@unimore.it; 2 Department of Biomedical Sciences for Health, University of Milan, 20133 Milan, Italy; rosario.caruso@unimi.it; 3 Health Professions Research and Development Unit, IRCCS Policlinico San Donato, 20097 San Donato Milanese, Italy; 4 Department of Biomedical, Metabolic and Neural Sciences, University of Modena and Reggio Emilia, 287 Giuseppe Campi Street, 41125 Modena, Italy; sergio.rovesti@unimore.it (S.R.); paola.ferri@unimore.it (P.F.)

**Keywords:** educational measurement, exploratory structural equation modeling, nursing education, psychometrics, reliability, student assessment, team-based learning, TBL-SAI, translation and validation, validity

## Abstract

**Background/Objectives**: Team-based learning is an educational strategy that promotes active learning and student engagement through structured team activities. It contrasts with traditional teaching models by emphasizing student preparation and collaboration. The TBL-SAI is a reliable and valid instrument designed to evaluate students’ attitudes towards TBL, assessing dimensions such as accountability, preference for lecture or team-based learning, and satisfaction with TBL. Validating the TBL-SAI in different languages ensures its applicability and accuracy in diverse educational contexts, supporting the global adoption of TBL methodologies. Thus far, no Italian validation of this tool has been performed. The aim of this study is to validate the Italian version of the Team-Based Learning Student Assessment Instrument (TBL-SAI) among nursing students. **Methods**: A methodological-driven translation process and a cross-sectional study design were used. The study was conducted from November 2022 to March 2023 and involved 202 nursing students from the University of Modena and Reggio Emilia (Italy). Convenience sampling was employed to recruit participants who had experienced at least one TBL session during the academic year 2022/2023. The validation process included translation, back-translation, and expert panel review, followed by a pilot test to ensure clarity and comprehension. Data were collected using the self-administered TBL-SAI. The responses were analyzed using Exploratory Structural Equation Modeling (ESEM) to assess the scale’s validity and McDonald’s ω to evaluate internal consistency. **Results**: The ESEM results supported the validity of the Italian TBL-SAI, maintaining the factor structure proposed in the original instrument. The model fit indices indicated a good fit (χ^2^(318) = 384.097, *p* = 0.0065; RMSEA = 0.032; CFI = 0.974; TLI = 0.957). McDonald’s ω values exceeded 0.70 for all factors, confirming adequate internal consistency. **Conclusions**: This study successfully validated the Italian version of the TBL-SAI, demonstrating its reliability and validity for assessing perceptions of TBL among Italian undergraduate nursing students. However, future studies should employ Confirmatory Factor Analysis to further test the proposed factor structure and explore the instrument’s applicability in various educational settings. The validated TBL-SAI is recommended for use in evaluating students’ attitudes towards TBL, providing actionable feedback for educators to improve teaching methods and integrate TBL methodologies effectively.

## 1. Introduction

Developed by Larry K. Michaelsen in the 1980s in response to increasing class sizes and unsatisfactory student involvement, team-based learning (TBL) has overturned the typical classroom structure [1]. In contrast to the traditional teaching model, where students are passive listeners and the teacher plays a central role, in TBL, students are actively engaged in the learning process while teachers act as facilitators [2]. Moreover, this instructional strategy supports the flipped classroom method [3], which promotes individual and group accountability. Students must come prepared for TBL sessions because, in class, they work within small groups to solve clinically relevant problems through the involvement of their peers [1]. Three sequential steps comprise the structured format of TBL: pre-class preparation, individual and team readiness assurance tests (RATs), and problem-solving activities [4].

In the pre-class preparation step, students are required to study pre-selected material in order to master the key concepts of the TBL topic. The second step can be divided into two parts. In the first one, known as the individual Readiness Assurance Test (iRAT), in a predetermined amount of time, students individually complete a test composed of 10 to 15 questions that are used to determine their understanding of the key concepts presented in the pre-class material. In the second part, the team Readiness Assurance Test (tRAT), the students complete the same test applied previously, but this time, they are within a team, the composition of which is determined in advance by the teacher. In the third step, working together in the same RAT groups, students are called upon to use their collective knowledge and clinical reasoning ability to solve challenging clinical problems that apply to real-life situations [1,2]. This collaborative effort aims to enhance their understanding of the subject matter and to foster critical thinking and teamwork skills by developing a deeper appreciation for the complexities of clinical decision-making required in real-world healthcare environments.

In recent years, considerable research has focused on identifying the benefits of TBL. Studies have revealed an increased sense of classroom engagement [5,6], the development of communication skills and self-directed learning [5,7], enhanced collaboration and teamwork skills [6], the development of critical and problem-solving skills [8], and a significant increase in academic knowledge and clinical performance [7,9]. Additionally, TBL has been shown to improve the motivation and satisfaction of students with the learning process, leading to better retention of course material [4,10]. Furthermore, the active learning environment promoted by TBL encourages continuous feedback and reflection, which are key components in developing lifelong learning habits [2].

Due to these advantages, TBL is gaining popularity worldwide as a form of active learning in health-related programs of study, such as medicine [11], nursing [6], physical therapy [12], dentistry [13], and pharmacy [14]. Furthermore, TBL could also be applied in an online context. The high level of interaction that this methodology requires was a particular strength during the COVID-19 pandemic, in which restrictions on social relationships strained educational systems worldwide. Studies conducted during those years showed that online TBL proved to be equally effective as the traditional face-to-face delivery mode [15,16].

In order to enable an increasingly widespread implementation of this teaching methodology within health professional curricula, it is important to have reliable and valid tools to assess students’ perception of TBL. The availability of such tools ensures the systematic evaluation of the efficacy of TBL in educational contexts and the improvements of TBL initiatives based on valid and reliable assessments. Several tools have been developed to assess teamwork and TBL. For example, the Extended Matching Questionnaire (EMQ) measures team performance in structured settings [17], while the Team-Q, developed to assess teamwork skills, has shown high internal consistency and utility in evaluating individual contributions to group tasks [18]. The Assessment of Teamwork in Undergraduate Education also provides a robust framework for evaluating individual teamwork skills across diverse disciplines [19]. Among these tools, the Team-Based Learning Student Assessment Instrument (TBL-SAI) stands out as a comprehensive and specialized instrument designed to evaluate students’ accountability, satisfaction, and preference for TBL versus lecture-based formats [20,21,22].

The TBL-SAI uniquely addresses the core components of TBL by measuring outcomes and critical pedagogical aspects that drive its success, and it was supported by evidence of validity and reliability [22]. Its multidimensional approach ensures a multifactorial understanding of how TBL impacts learning experiences, making it particularly valuable for educators and researchers aiming to optimize TBL practices. While the TBL-SAI has been translated into multiple languages, psychometric validation has only been conducted for the Persian, Arabic, and Brazilian versions [23,24,25]. Initially tested on a large sample of undergraduate nursing students (n = 396), the TBL-SAI has also undergone validity and reliability evaluations in pharmacy students [26] as well as in medical, nursing, and midwifery students [27].

Thus far, the TBL-SAI has proven to be effective in assessing students’ attitudes towards TBL. Despite the availability of other instruments, the TBL-SAI remains distinctive in its comprehensive assessment of key dimensions of TBL [22]. Although the number of nursing programs adopting TBL is growing in Italy, there is a significant gap in research regarding validating an Italian version of the TBL-SAI. Sound psychometric validation is pivotal to precisely measuring students’ opinions in Italy regarding their TBL experience and enabling cross-national research. Addressing the lack of evidence regarding the Italian TBL-SAI version is crucial for enhancing Italian educational practices. Also, it holds international significance by facilitating comparative studies and contributing to the global body of research on TBL efficacy. Therefore, this study aimed to translate and validate the Italian version of the TBL-SAI among undergraduate nursing students.

## 2. Materials and Methods

### 2.1. Design

The study comprised two main parts. The first part was methodological and focused on translating the TBL-SAI into Italian. The second part involved data collection via a cross-sectional study to validate the translated instrument. The reporting of the cross-sectional part of the study followed the “Strengthening the Reporting of Observational Studies in Epidemiology” (STROBE) statement [28].

### 2.2. Setting and Sample

Convenience sampling was used to recruit participants. The inclusion criteria were: (1) students enrolled in the first and second year of the Bachelor of Science in Nursing program at the University of Modena and Reggio Emilia in Modena, Italy, during the academic year (AY) 2022/23; (2) students who participated in at least one in-person TBL session held during the AY 2022/23; (3) voluntarily agreed to participate in this study. No exclusion criteria were set up, as the aim was to include all eligible students to obtain a comprehensive and representative sample for validating the Italian version of the TBL-SAI.

For this study, the minimum sample size was calculated based on the formula provided by Westland (2010) for determining lower bounds on sample size in structural equation modeling. The parameters used for this calculation were: Number of Observed Variables (j) = 33; Number of Latent Variables (k) = 7; Anticipated Effect Size (δ) (i.e., the strength of the relationship between the variables) = 0.4 indicates a moderate strength; Type I Error Rate (α) = 0.05; and Desired Statistical Power (1 − β) = 0.80. Using these parameters, we applied the formula for the lower-bound sample size:

(1) $$N\geq \frac{\left(j-k+1\right)z_{\left\{1-\frac{\alpha }{2}\right\}}^{2}}{\delta^{2}}$$

From this calculation, the minimum sample size was determined to be 170 respondents. To further validate this minimum sample size and to perform a sensitivity analysis, a simulation was conducted using the “lavaan” package in R version 4.2.2 (R Core Team, 2023) (see Supplementary File S1).

The SEM model specified included various latent constructs such as Contribution to Team (CTT), Preparation (PREP), Accountability (ACC), Team-based Learning (TBL), Lecture (LEC), Preference (PREF), and Satisfaction (SAT). The simulation in R indicated that while a sample size of 170 was the minimum required, a larger sample size would provide more robust power for detecting the effects. Specifically, a sample size greater than 200 might be more accurate to achieve the desired statistical power. Based on the simulation and, considering practical constraints and aiming for a representative sample, the minimum sample size of 170 was set as the target for this study, with an optimal target of 250 respondents to ensure robustness in the findings.

### 2.3. The Measurement Instrument

The TBL-SAI is a self-administered instrument comprising 33 items developed to measure students’ attitudes towards TBL [22]. The author of the original scale, Heidi Mennenga, granted permission to adapt and test the tool in the Italian setting. The instrument is composed of three subscales: (a) accountability, intended as student pre-class preparation and contribution to the team success (items 1 to 8, scores from 8 to 40), which encompasses two factors: contribution to team and preparation; (b) preference for lecture versus TBL, in terms of student ability to recall material and level of attention (items 9 to 24, scores from 16 to 80), which has two factors: team-based learning and lecture (i.e., student ability to recall material of the lecture); (c) student satisfaction with TBL (items 25 to 33, scores from 9 to 45), which is unidimensional. Overall, the TBL-SAI has a total score that ranges from 33 to 165.

Scores above 99 on the total scale, >24 for the accountability subscale, >48 for the subscale of Preference for TBL over lecture, and >27 for the subscale of student satisfaction indicate a positive perception of using TBL.

The participants were asked to respond using a five-point Likert scale ranging from 1 to 5 (1 = strongly disagree, 2 = disagree, 3 = neutral, 4 = agree, 5 = strongly agree). Ten items with negative wording were reverse-scored from 5 to 1. Demographic data (gender, age, highest level of education attained) were also collected.

The TBL-SAI was originally validated by involving nearly 400 students in an American Bachelor of Science in Nursing program in 2009/2010. By achieving an overall Cronbach’s Alpha score of 0.941 and scores of 0.782, 0.893, and 0.942, respectively, in the three subscales, the TBL-SAI proved to be a valid and reliable tool for accurately measuring students’ perceptions toward TBL.

#### Translation Process

The TBL-SAI was translated from English into Italian by two independent researchers (PF and VV) who were native to Italian but fluent in English and knew the TBL methodology and the questionnaire. After reviewing and reaching a consensus between the translated versions, a unique Italian version of the instrument was provided. After that, two native English speakers, without knowledge of the original instrument, translated the forward version back into English. These versions were compared with the original questionnaire. Later, an expert panel consisting of nursing educators with experience in using TBL reviewed and confirmed the final version.

Finally, a pilot study was conducted with a convenience sample of 30 undergraduate nursing students from a Bachelor of Science in Nursing program at another university in the same region. To test the clarity and understandability of each item, respondents were asked to paraphrase each sentence in written form to see whether or not it was understood. In this way, the Italian version of the TBL-SAI was developed.

### 2.4. Data Collection

Data were collected from November 2022 to March 2023. Participants were given an explanation of the study’s purpose and procedure in both oral and written form, and informed consent was obtained. They were informed that their participation was entirely voluntary and that all information would be kept confidential. Data were gathered after students had completed the last TBL session of the AY, and it took approximately 10 min to collect it. Questionnaire forms were distributed by the researchers to the students who completed them under supervision. A total of 204 questionnaires were returned (response rate 70.3%). In addition to the TBL-SAI, the information collected aimed to describe the sample were: sex (male, female, prefer not to respond), age (years), year of the course, and the number of attended TBL activities.

### 2.5. Data Analysis

The data were assessed for distribution and missingness and summarized to describe the sample characteristics according to the nature of the variables and their distribution. An Exploratory Structural Equation Modeling (ESEM) was performed to determine the psychometric validity using Mplus 8.1 (Los Angeles, CA, USA: Muthén & Muthén).

The dimensionality of the TBL-SAI was initially and preliminarily explored using a scree plot generated from the eigenvalues of the item correlation matrix. The scree plot supported the retention of three factors, aligning with the theoretical structure proposed by Mennenga [22]. While the scree plot provided initial evidence for the dimensionality of the scale, its limitations in detecting higher-order factors necessitated further validation through more sophisticated analyses. In this context, the decision to use ESEM was guided by recent recommendations to perform construct validation by employing factor analyses [29]. The choice of ESEM was guided by its ability to provide a more flexible and parsimonious representation of the factor structure compared to traditional confirmatory factor analysis (CFA). ESEM allows for modeling complex relationships between factors, including cross-loadings, often present in educational and psychological data. This flexibility is particularly important in the context of the TBL-SAI, where the subscales encompass multiple dimensions that may overlap. In the case of the Italian validation of the TBL-SAI, ESEM was found to be the most appropriate because it more accurately captured the relationships between the subscales and their underlying factors, considering that Exploratory Factor Analysis (EFA) may not be adequate to assess high-dimensionality factors as per the case of contribution to team and preparation, both predicted by Accountability (higher-order factor) and considering team-based learning and lecture, both predicted by a second-order factor (i.e., preference for lecture versus TBL). CFA was not adequate as well because the dimensionality of the scale was never assessed in the Italian context. Therefore, ESEM was the most suitable approach for psychometrically validating the Italian version of the TBL-SAI [30].

The model was constructed to accurately mirror the theoretical structure of the TBL-SAI and offer the best fit for the data. The Unweighted Least Squares Mean and Variance adjusted (ULSMV) estimator, chosen for its unique ability to provide robust estimates for models with ordinal indicators, was employed [31]. The model utilized a targeted rotation method, a feature that simplified the factor structure by rotating the factors to a target pattern, thereby facilitating a clearer and more interpretable solution. The model’s specification adhered to the previously described dimensionality of the scale: Contribution to Team and Preparation (first-order factors) were elucidated by items of Accountability (hypothetical second-order factor behavior, the first subscale), Team-Based Learning and Lecture (first-order factors) were clarified by Preference (hypothetical second-order factor behavior, the second subscale), and Satisfaction was a unidimensional factor (third subscale). In essence, the current analysis on Accountability and Preference laid the groundwork for testing their behaviors as second-order factors in future research with new samples. In this analysis, it was possible to demonstrate their behavior in predicting the observed variables of their theoretical lower-order factors. The model allowed Accountability, Preference, and Satisfaction to correlate with each other. The estimates of the model were standardized for the reporting, and the estimated variance-covariance matrix of the latent variables and modification indices, which suggest potential improvements to the model, were examined.

The fit of the ESEM model was evaluated using the following indices, each of which played a crucial role in assessing the model’s performance: the Chi-Square Test of Model Fit (χ^2^), which examined the discrepancy between the observed and model-implied covariance matrices (a non-significant chi-square indicates a good fit, although this index is highly influenced by sample size); Root Mean Square Error of Approximation (RMSEA), which gauged the error of approximation per degree of freedom in the model (values less than 0.06 indicate a good fit); the Comparative Fit Index (CFI), and the Tucker–Lewis Index (TLI), which compared the fit of the target model to a baseline model (values higher than 0.90 are excellent).

Convergent and discriminant validity were assessed through inter-factor correlations. Evidence of discriminant validity was supported by moderate correlations between the subscales, demonstrating that each subscale captured unique constructs as predicted by the theoretical framework of the TBL-SAI [32]. This approach aligns with established guidelines for assessing construct validity in educational and psychological research and provides preliminary theory-based evidence of convergent and discriminant validity [33]. The hypotheses guiding this analysis are grounded in the theoretical framework of TBL-SAI. A positive and moderate-to-strong correlation between Satisfaction and TBL was expected, as these constructs are closely linked through their shared emphasis on student engagement and active learning. Similarly, Accountability and Preparation were hypothesized to show a strong positive relationship, reflecting their theoretical overlap, as accountability inherently includes preparation for TBL sessions. 

In contrast, a negative correlation was anticipated between Lecture-Based Learning and Satisfaction, as lecture-based methods are fundamentally different from the collaborative and interactive nature of TBL. Furthermore, weak or non-significant correlations were expected between Preference for Lecture vs. TBL and Contribution to Team, given that these constructs address distinct dimensions: individual preferences versus teamwork contributions. These hypotheses were developed to reflect the TBL-SAI framework, which posit both interconnected and distinct relationships between its subscales, thus supporting convergent and discriminant validity.

Reliability was established by calculating McDonald’s ω for each subscale (values above 0.70 indicate adequate internal consistency). Statistical significance was set with an alpha equal to 5%.

## 3. Results

### 3.1. Translation and Back-Translation Results

The back-translation process revealed no discrepancies between the back-translated versions and the original questionnaire, confirming the semantic and conceptual alignment of the Italian version with the original TBL-SAI. The expert panel of nursing educators (n = 6) validated the equivalence of the translated items with the original instrument and endorsed the final version.

In the pilot test conducted with 30 undergraduate nursing students, all items were reported to be clear and comprehensible. Participants successfully paraphrased each item, demonstrating that the translated items were well understood. No further modifications were required, indicating that the Italian version of the TBL-SAI was ready for validation in a broader sample.

### 3.2. Sample Characteristics

Table 1 presents the characteristics of the students who participated in the study. The sample consisted of 202 students, with a predominance of females (80.2%). The median age of the participants was 21 years, with an interquartile range (IQR) of 20 to 22 years and an overall age range of 18 to 53 years.

Regarding the academic year, 30.9% of the students were in their first year, while 69.1% were in their second year. Regarding participation in Team-Based Learning (TBL) activities, 9.3% of the students attended one activity, 61.8% attended two activities, and 28.9% attended three activities.

### 3.3. Construct Validity: ESEM

**Figure 1:**
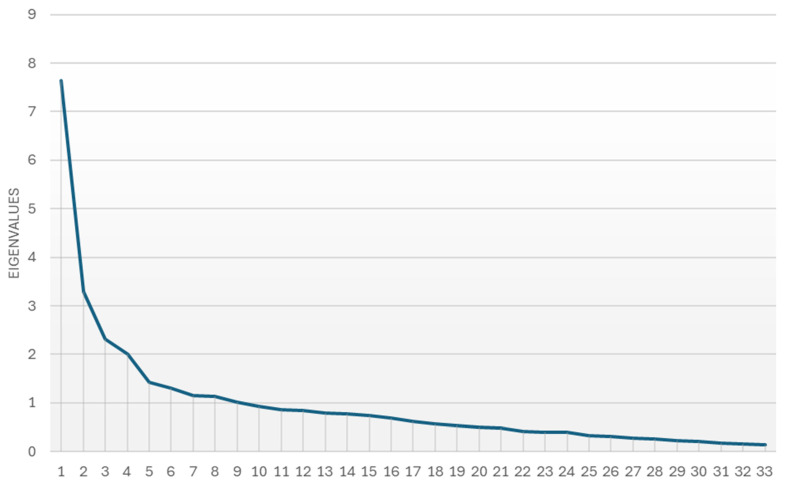
A scree plot was generated from the item correlation matrix’s eigenvalues.

As a preliminary step to the ESEM, a scree plot was generated from the item correlation matrix’s eigenvalues to investigate the TBL-SAI’s dimensionality (Figure 1). The scree plot showed a distinct “elbow” after the third factor, suggesting that three factors should be retained. Another plausible interpretation could suggest considering the “elbow” after the fifth factor. These findings are consistent with the theoretical framework of the TBL-SAI, which comprises three main subscales: Accountability, Preference, and Satisfaction and also considering additional factors (e.g., elbow given by the fifth factor) could be consistent with the idea of a more complex factor structure. While the scree plot could support the framing of hypotheses regarding the overall dimensionality of the instrument, it did not account for higher-order factors or cross-loadings. Thus, ESEM was employed to provide a more robust and flexible validation of the scale’s factor structure.

The fit of the ESEM model was very satisfactory in explaining sample statistics: χ^2^(318) = 384.097, p = 0.0065; RMSEA = 0.032, 90%CI: 0.018-0.043; CFI = 0.974; TLI = 0.957. Details of the model are shown in Table 2.

For the Contribution to Team factor, items 1, 2, and 3 emerged as significant. Factor loadings for these items ranged from 0.398 to 0.765, with standard errors (SE) between 0.085 and 0.091. In the Preparation factor, items 4, 5, 6, 7, and 8 were included as significant. Factor loadings for these items ranged from 0.373 to 0.664, with SEs ranging from 0.080 to 0.102. The Accountability factor included items 1 to 8, indicating that it may behave as a second-order factor that could predict Contribution to Team and Preparation. Factor loadings for items 1 to 8 ranged from 0.390 to 0.878, with SEs ranging from 0.072 to 0.107.

The Team-Based Learning factor included items 11, 13, 14, 15, 17, 19, 20, and 23. Factor loadings for these items ranged from 0.334 to 0.884, with SEs ranging from 0.058 to 0.140. For the Lecture factor, items 9, 10, 12, 16, 18, 21, 22, and 24 were included. Factor loadings for these items ranged from 0.325 to 0.682, with SEs ranging from 0.079 to 0.316. The Preference for Lecture versus TBL factor predicted the same Team-Based Learning and Lecture items, behaving as a plausible second-order factor, including items 9 to 24. Factor loadings for these items ranged from 0.305 to 0.671, with SEs ranging from 0.102 to 0.410.

The Satisfaction factor included items 25 to 33. Factor loadings for these items ranged from 0.361 to 0.980, with SEs ranging from 0.041 to 0.093.

### 3.4. Inter-Factor Correlations

**Figure 2:**
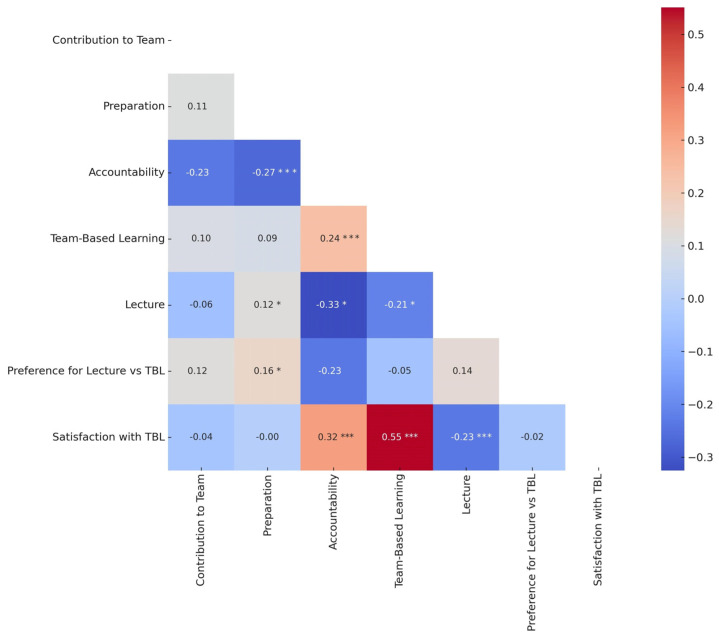
Correlogram and heatmap of inter-factor correlations. * indicates *p* < 0.05, *** indicates *p* < 0.001.

Inter-factor correlations among the TBL-SAI subscales were examined to evaluate discriminant validity. The correlogram (Figure 2) provides a visual representation of these relationships, including their significance levels.

As hypothesized, a strong positive relationship was observed between Satisfaction and Team-Based Learning (r = 0.55, *p* < 0.001), supporting the theoretical link between these constructs, both of which emphasize active and engaging learning experiences. Similarly, the moderate correlation between Accountability and Satisfaction (r = 0.32, *p* < 0.001) aligns with the hypothesis that higher perceived accountability, which reflects preparation and contribution to the team, enhances satisfaction in TBL settings.

As anticipated, a negative but significant correlation was found between Lecture-Based Learning and Satisfaction (r = −0.23, *p* = 0.001), supporting the hypothesis that preferences for lecture-based methods inversely affect satisfaction with TBL, as these two approaches represent contrasting educational philosophies. Other correlations, such as between Preference for Lecture vs. TBL and Preparation (r = 0.16, *p* = 0.014) or Accountability and Preparation (r = −0.27, *p* = 0.001), were significant and aligned with theoretical expectations, further supporting the framework.

In line with the hypotheses, weak or non-significant relationships were observed, such as between Preference for Lecture vs. TBL and Contribution to Team (r = 0.12, *p* = 0.251). These results highlight the distinctiveness of the subscales, demonstrating that they measure unique yet theoretically related constructs consistent with the theoretical assumptions underpinning the TBL-SAI.

### 3.5. Reliability

The Contribution to Team subscale had an ω of 0.710, while the Preparation subscale showed an ω of 0.755. The overall Accountability factor, which encompasses Contribution to Team and Preparation items, had an ω of 0.726.

For the Team-Based Learning subscale, the ω was 0.784; for the Lecture subscale, it was 0.796. The combined Preference for Lecture versus TBL factor, which includes items from both Team-Based Learning and Lecture, had an ω of 0.744. Finally, the Satisfaction subscale demonstrated the highest reliability with an ω of 0.878.

### 3.6. Scores of the TBL-SAI

Table 3 presents the descriptive statistics for the TBL-SAI scores, including the median, interquartile range (IQR), and skewness for the overall scale and its subscales.

The overall TBL-SAI score had a median of 133.5, an IQR of 125 to 140, and a skewness of −0.309, indicating a slight left skew. The Accountability subscale had a median score of 33 with an IQR of 31 to 36 and a skewness of −0.889, suggesting a more pronounced left skew. Within the Accountability subscale, the Contribution to the Team factor had a median of 13 with an IQR of 12.0 to 14 and a skewness of −1.144, while the Preparation factor had a median of 21 with an IQR of 19 to 23 and a skewness of −0.942.

For the Preference for Lecture versus TBL subscale, the median score was 60, with an IQR of 56 to 64 and a skewness of 0.214, indicating a slight right skew. This subscale includes the Team-Based Learning factor, which had a median score of 35, an IQR of 32 to 38, and a skewness of −0.677, and the Lecture factor, with a median score of 26, an IQR of 22 to 29, and a skewness of −0.234.

The Satisfaction with TBL subscale showed a median score of 40.5, an IQR of 36.25 to 43.75, and a skewness of −1.258, indicating a significant left skew.

## 4. Discussion

The translation and psychometric validation of the TBL-SAI into Italian provides a crucial tool for evaluating the perceptions regarding TBL among Italian undergraduate nursing students. This study is the first to use ESEM to assess the scale’s validity, paving the way for more analyses in educational research that allow researchers to measure the construct that the scale proposes to determine in a valid and reliable way [34]. Using ESEM, following an initial hypothesis-generating interpretation of the scree test obtained from the item correlation matrix’s eigenvalues, confirms the reliability and validity of the Italian version of the TBL-SAI. Also, it enhances the instrument’s applicability at an international level, paving the way for analyzing the complex dimensionality of the TBL-SAI in a standardized manner.

The results of this study enriched previous validations of the TBL-SAI in different languages, such as Persian, Arabic, and Brazilian Portuguese [23,24,25]. The fit of the ESEM model suggests that the TBL-SAI maintains its validity in Italian, keeping the same factor structure proposed during the developmental study [22], reinforcing its utility as a robust instrument for assessing students’ perceptions of TBL. In addition, as confirmed by McDonald’s ω, values exceeding 0.70 for all factors indicate adequate internal consistency. These results highlight the instrument’s reliability in measuring TBL. In particular, the three main factors—Accountability, Preference for Lecture versus TBL, and Satisfaction with TBL—remain consistent with the original tool [22]. Accountability encompasses Contribution to Team and Preparation, which reflect students’ responsibility and engagement in team-based learning activities. Preference for Lecture versus TBL evaluates students’ attitudes toward and preference for team-based versus traditional lecture-based teaching methods. Satisfaction captures students’ overall enjoyment and positive experiences with TBL. These factors, retained in their original conceptual form, ensure that the Italian version accurately measures the same constructs as the original tool.

Satisfaction with TBL: These results support its broader application in international educational research [23,24,25]. The ability of the TBL-SAI to consistently measure key constructs such as Accountability, Preference, and Satisfaction highlights its utility for comparative studies, enabling educators and researchers to assess the impact of TBL on student perceptions across diverse educational settings.

The validated Italian version of the TBL-SAI offers two major practical benefits for educational institutions and educators. Firstly, the instrument provides a reliable measure for evaluating students’ attitudes towards TBL, enabling educators to gather actionable feedback to improve teaching methods. This is particularly important given the increasing adoption of TBL in various educational settings, as highlighted by previous studies that have demonstrated its effectiveness in enhancing student engagement, communication skills, and critical thinking abilities [5,6,8].

Secondly, insights from the TBL-SAI could guide the integration and optimization of TBL methodologies within nursing curricula. Educators could tailor their teaching strategies to better align with learning needs by understanding students’ preferences and experiences. This possibility aligns with recent findings [3,7,35], which emphasized the importance of adapting educational approaches to meet the specific needs of students in health-related programs. In particular, some previous research has shown that TBL could significantly improve academic knowledge and clinical performance [7,35], and having a standardized assessment tool enables more rigorous evaluations and comparisons of these outcomes internationally.

In educational measurement, it is crucial to use valid and reliable tools to accurately capture students’ perceptions and experiences, as these insights directly inform teaching practices and curriculum development [36]. Valid measures ensure that the data collected indeed represents the constructs being studied. In the context of TBL, valid instruments can accurately measure students’ attitudes, engagement, and satisfaction with the learning process. In addition, such measures provide consistency in results, which is fundamental for longitudinal studies and comparative research. This consistency allows educators and researchers to track changes over time and make comparisons across different groups or educational settings.

Future research needs to conduct longitudinal studies to track changes in students’ perceptions of TBL over time and their impact on academic performance and clinical skills. This will allow researchers to uncover the interplay of several mechanisms related to TBL per se and the students’ characteristics to inform innovative strategies better to optimize the efficacy of TBL. Moreover, future research should also utilize the hypothetical factor structure established by this study, which proposed that two of the three subscales are second-order factors. This necessitates new sampling and a CFA to validate this proposed structure. Such efforts will help refine the TBL-SAI and ensure its robustness and applicability in various educational contexts. In addition, evidence of construct validity using criterion-based approaches is needed to strengthen the current base of validity for this tool, according to the “Consensus-based Standards for the Selection of Health Measurement Instruments” (COSMIN) standards [37].

### Limitations

While the study successfully met its objectives, several limitations should be acknowledged. The study sample was drawn from a single university, which may need to fully capture the diversity of the student population across Italy. This limits the generalizability of the findings to other educational settings within the country. Another limitation to the generalizability was the presence of students attending only the first or second year of the undergraduate program. This limitation is justified by the faculty’s decision to gradually resume the use of TBL after its suspension during the COVID-19 period. Although TBL was already in use before the pandemic period, its adoption had been suspended due to the impossibility of reconciling the restrictions imposed by the authorities with the face-to-face methodology. When in A.A. 2021/22, the university authorized all teaching activities to take place in presence, it was decided to adopt TBL again with the first and second years to allow students and lecturers to familiarize themselves with the method.

In addition, the study’s cross-sectional design restricts the ability to infer causality or observe changes in students’ perceptions over time. Longitudinal studies would be necessary to understand how these perceptions evolve. Furthermore, the study focused exclusively on nursing programs, limiting the tool’s evaluation in other educational contexts. Expanding its application to a broader range of disciplines could enhance its versatility and confirm its adaptability to diverse educational settings.

Given the self-administered nature of the questionnaire, there is a risk of socially desirable responses, which could affect the accuracy of the data collected and require further investigations. While initial convergent and discriminant validity were examined through inter-factor correlations, no further tests of convergent and discriminant validity were performed. Future research should include more advanced validity tests and comparisons with external constructs to strengthen evidence of construct validity.

Despite these limitations, this study makes a valuable contribution to the field of educational research by supporting the global adoption of TBL strategies. The validation of the TBL-SAI in Italian adds to its growing body of evidence as a reliable and adaptable tool for assessing students’ perceptions of TBL in diverse cultural and educational contexts.

## 5. Conclusions

This study successfully validated the Italian version of the TBL-SAI, demonstrating evidence of its validity and reliability. Using ESEM, we confirmed the instrument’s factor structure. We provided additional nuances of the scale dimensionality, making TBL-SAI a valuable tool for assessing perceptions of TBL among Italian undergraduate nursing students. Future studies are needed to test the proposed factor structure with CFA further, explore the instrument’s applicability in different educational settings and student populations, and test measurement invariance with other cultural contexts. We advise using this tool to assess students’ attitudes towards TBL, to gather actionable feedback for improving teaching methods, and to guide the integration and optimization of TBL methodologies within nursing and other health-related curricula.

## Figures and Tables

**Table 1:** Characteristics of the students.

Variables	Subtype	N	Percentage	Median, IQR ^1^
Gender	Female	162	80.2	
	Male	40	19.8	
Age	-	-	-	21, 20–22 (range 18–53)
Year of the Course	First	63	30.9	
	Second	141	69.1	
Number of attended TBL activities	One activity	19	9.3	
	Two activities	126	61.8	
	Three activities	59	28.9	

Notes: ^1^ IQR—interquartile range.

**Table 2:** Estimates of the ESEM.

	CTT ^1^ (Loading, SE ^8^) Lower-Order Factor	Prep ^2^ (Loading, SE) Lower-Order Factor	Acc ^3^ (Loading, SE) Hypothetical Second-Order Factor	TBL ^4^ (Loading, SE) Lower-Order Factor	Lec ^5^ (Loading, SE) Lower-Order Factor	Pref ^6^ (Loading, SE) Hypothetical Second-Order Factor	Sat ^7^ (Loading, SE) Lower-Order Factor
Item1	**0.722**, 0.085	0.068, 0.072	**0.390**, 0.107	0.084, 0.067	−0.051, 0.055	−0.009, 0.060	−0.047, 0.059
Item2	**0.765**, 0.091	−0.061, 0.052	**0.583**, 0.101	−0.179, 0.055	−0.091, 0.048	−0.041, 0.043	0.148, 0.053
Item3	**0.398**, 0.088	0.193, 0.090	**0.560**, 0.072	0.113, 0.079	−0.046, 0.074	−0.044, 0.078	−0.019, 0.075
Item4	−0.156, 0.066	**0.664**, 0.102	**0.612**, 0.080	0.080, 0.085	−0.181, 0.075	−0.073, 0.098	−0.098, 0.082
Item5	0.049, 0.065	**0.455**, 0.080	**0.547**, 0.080	−0.033, 0.087	0.104, 0.072	0.069, 0.076	−0.039, 0.080
Item6	0.197, 0.046	**0.593**, 0.101	**0.878**, 0.075	0.173, 0.057	0.153, 0.050	−0.004, 0.057	−0.151, 0.056
Item7	−0.124, 0.058	**0.373**, 0.087	**0.564**, 0.083	−0.062, 0.065	−0.123, 0.057	−0.021, 0.075	0.257, 0.065
Item8	−0.044, 0.061	**0.625**, 0.089	**0.685**, 0.087	−0.079, 0.070	0.013, 0.067	0.096, 0.062	0.128, 0.071
Item9	0.035, 0.052	−0.188, 0.052	0.028, 0.053	−0.089, 0.046	**0.659**, 0.260	**0.521**, 0.410	−0.025, 0.048
Item10	−0.088, 0.056	−0.139, 0.072	0.143, 0.045	−0.070, 0.044	**0.628**, 0.316	**0.671**, 0.400	0.037, 0.051
Item11	−0.046, 0.108	−0.195, 0.092	0.217, 0.089	**0.367**, 0.109	−0.020, 0.069	**0.317**, 0.104	0.032, 0.084
Item12	−0.108, 0.063	−0.023, 0.074	0.081, 0.066	0.102, 0.067	**0.593**, 0.167	**0.306**, 0.338	0.030, 0.069
Item13	−0.035, 0.088	−0.270, 0.078	0.176, 0.081	**0.337**, 0.140	−0.034, 0.069	**0.365**, 0.102	0.264, 0.072
Item14	−0.015, 0.102	−0.314, 0.082	0.035, 0.086	**0.334**, 0.126	−0.105, 0.071	**0.397**, 0.145	0.123, 0.085
Item15	−0.048, 0.068	0.197, 0.078	0.099, 0.070	**0.560**, 0.074	−0.034, 0.076	**0.342**, 0.146	0.168, 0.064
Item16	−0.031, 0.070	−0.155, 0.108	−0.062, 0.074	−0.077, 0.104	**0.509**, 0.134	**0.464**, 0.298	−0.004, 0.079
Item17	0.009, 0.050	0.142, 0.060	0.027, 0.060	**0.790**, 0.058	−0.074, 0.054	**0.318**, 0.214	−0.055, 0.057
Item18	−0.009, 0.086	0.402, 0.104	−0.122, 0.076	−0.129, 0.070	**0.682**, 0.194	**0.401**, 0.351	0.169, 0.068
Item19	−0.006, 0.048	0.070, 0.067	0.030, 0.050	**0.884**, 0.066	−0.027, 0.054	**0.370**, 0.201	−0.024, 0.047
Item20	0.003, 0.052	−0.061, 0.045	−0.046, 0.057	**0.726**, 0.059	−0.008, 0.057	**0.343**, 0.164	0.195, 0.051
Item21	0.072, 0.084	0.046, 0.097	0.052, 0.093	0.089, 0.098	**0.330**, 0.079	**0.305**, 0.163	−0.107, 0.093
Item22	0.063, 0.090	−0.112, 0.085	0.118, 0.086	0.175, 0.094	**0.325**, 0.141	**0.367**, 0.153	−0.109, 0.094
Item23	0.019, 0.061	0.000, 0.061	−0.084, 0.053	**0.378**, 0.066	−0.042, 0.073	**0.368**, 0.119	0.456, 0.060
Item24	0.010, 0.061	0.046, 0.075	−0.146, 0.063	0.166, 0.073	**0.505**, 0.129	**0.305**, 0.256	−0.125, 0.073
Item25	0.033, 0.048	−0.020, 0.065	0.059, 0.066	−0.158, 0.070	−0.039, 0.054	0.001, 0.054	**0.980**, 0.041
Item26	−0.079, 0.065	0.111, 0.066	0.026, 0.072	0.250, 0.080	0.007, 0.065	0.103, 0.080	**0.617**, 0.058
Item27	0.075, 0.062	0.162, 0.065	0.042, 0.055	0.142, 0.069	−0.091, 0.054	0.002, 0.100	**0.764**, 0.046
Item28	−0.305, 0.081	−0.021, 0.093	0.073, 0.087	0.050, 0.104	0.007, 0.094	−0.154, 0.080	**0.361**, 0.084
Item29	−0.115, 0.053	0.022, 0.076	0.043, 0.061	−0.083, 0.063	−0.045, 0.064	0.053, 0.051	**0.867**, 0.053
Item30	−0.019, 0.086	−0.106, 0.106	0.119, 0.089	0.235, 0.122	0.067, 0.144	−0.320, 0.073	**0.460**, 0.093
Item31	0.110, 0.073	0.015, 0.092	−0.233, 0.062	0.351, 0.081	0.006, 0.061	0.076, 0.093	**0.509**, 0.066
Item32	0.081, 0.060	−0.034, 0.075	0.021, 0.053	0.020, 0.068	−0.009, 0.063	−0.064, 0.061	**0.810**, 0.057
Item33	0.179, 0.071	−0.096, 0.088	0.073, 0.068	0.177, 0.079	0.106, 0.088	0.123, 0.075	**0.683**, 0.067

Notes: ^1^ CTT—Contribution to Team; ^2^ Prep—Preparation; ^3^ Acc—Accountability; ^4^ TBL—Team-Based Learning; ^5^ LEC—Lecture; ^6^ Pref—Preference for Lecture versus TBL; ^7^ SAT—Student Satisfaction with TBL; ^8^ SE—standard error. The estimates provided in the table represent a fully standardized solution. Bold values indicate estimates greater than 0.31. Items were retained in factors based on theoretical alignment and empirical evidence. The theoretical structure included: (a) CTT and Prep as components of Acc. CTT was represented by Item1 to Item3, while Prep was represented by Item4 to Item8. (b) TBL and LEC as components of Pref. TBL was represented by items that explicitly aligned with team-based learning (Item11, Item13–15, Item17, Item19–20, and Item23), while LEC was represented by items related to lecture-based learning (Item9–10, Item12, Item16, Item18, Item21–22, and Item24). Items not relevant to these domains were fixed at zero. (c) SAT was treated as a unidimensional construct represented by Item 25 to Item 33, with items from other subscales excluded from this factor. Items were assigned to factors based on their primary loadings (≥0.40) while considering cross-loadings to ensure consistency with theoretical assumptions. (d) Hypothetical second-order factors (Acc and Pref) were modeled to reflect their predicted relationships with lower-order factors (CTT, Prep, TBL, and LEC). These relationships remain hypothetical, requiring further empirical validation through CFA in an independent sample. (e) Cross-loadings were expected in this ESEM and were evaluated to account for multidimensionality (lower and higher factors) while preserving the theoretical integrity of the model. In other words, items were expected to be predicted by their first- and second-level factors.

**Table 3:** Scores of the TBL-SAI.

TBL-SAI Scores	Median	IQR ^1^	Skewness
Overall	133.5	125–140	−0.309
Accountability	33	31–36	−0.889
Contribution to team	13	12.0–14	−1.144
Preparation	21	19–23	−0.942
Preference for lecture versus TBL ^2^	60	56–64	0.214
Team-based learning	35	32–38	−0.677
Lecture	26	22–29	−0.234
Satisfaction with TBL	40.5	36.25–43.75	−1.258

Notes: ^1^ IQR—interquartile range; ^2^ TBL—team-based learning.

## Data Availability

Detailed data are available upon reasonable request to the corresponding author.

## Supplementary Materials

The following supporting information can be downloaded at: https://www.mdpi.com/article/10.3390/nursrep15010026/s1, File S1: Power analysis.

## Public Involvement Statement

There was no public involvement in any aspect of this research.

## Guidelines and Standards Statement

This manuscript was drafted against the STROBE guidelines for cross-sectional studies.

## References

[1] Burgess A., van Diggele C., Roberts C., Mellis C. (2020). Team-Based Learning: Design, Facilitation and Participation. BMC Med. Educ..

[2] Parmelee D., Michaelsen L.K., Cook S., Hudes P.D. (2012). Team-Based Learning: A Practical Guide: AMEE Guide No. 65. Med. Teach..

[3] Jakobsen K.V., Knetemann M. (2017). Putting Structure to Flipped Classrooms Using Team-Based Learning. Int. J. Teach. Learn. High. Educ..

[4] Haidet P., Levine R.E., Parmelee D.X., Crow S., Kennedy F., Kelly P.A., Perkowski L., Michaelsen L., Richards B.F. (2012). Perspective: Guidelines for Reporting Team-Based Learning Activities in the Medical and Health Sciences Education Literature. Acad. Med..

[5] Alberti S., Motta P., Ferri P., Bonetti L. (2020). The Effectiveness of Team-Based Learning in Nursing Education: A Systematic Review. Nurse Educ. Today.

[6] Considine J., Berry D., Allen J., Hewitt N., Oldland E., Sprogis S.K., Currey J. (2021). Team-Based Learning in Nursing Education: A Scoping Review. J. Clin. Nurs..

[7] Gao X., Yan D., Zhang Y., Ruan X., Kang T., Wang R., Zheng Q., Chen S., Zhai J. (2024). Comparison of the Impact of Team-Based Learning and Lecture-Based Learning on Nursing Students’ Core Competencies: A Systematic Review and Meta-Analysis. Nurse Educ. Pract..

[8] Yeung M.M.-Y., Yuen J.W.-M., Chen J.M.-T., Lam K.K.-L. (2023). The Efficacy of Team-Based Learning in Developing the Generic Capability of Problem-Solving Ability and Critical Thinking Skills in Nursing Education: A Systematic Review. Nurse Educ. Today.

[9] Zhang Q., Tang X., Zhao Y., Wang Z. (2023). Team-Based Learning vs. Lecture-Based Learning in Nursing: A Systematic Review of Randomized Controlled Trials. Front. Public Health.

[10] Koles P.G., Stolfi A., Borges N.J., Nelson S., Parmelee D.X. (2010). The Impact of Team-Based Learning on Medical Students’ Academic Performance. Acad. Med..

[11] Sterpu I., Herling L., Nordquist J., Rotgans J., Acharya G. (2024). Team-Based Learning (TBL) in Clinical Disciplines for Undergraduate Medical Students-a Scoping Review. BMC Med. Educ..

[12] Yaqoob M.F., Khalid Z., Azim M.E., Ahsan S., Hassan M.F., Naeem A. (2021). Perceptions Regarding Team-Based Learning among Undergraduate Physical Therapy Students. J. Pak. Med. Assoc..

[13] James Trill B., Panesar B., Dave M., Vahid Roudsari R., Javidi H. (2024). Is Team-Based Learning an Alternative Approach for UK Undergraduate Dental Education? A Scoping Review of the Literature. Br. Dent. J..

[14] Korayem G.B., Alghamdi A.A., Aljuhani O., Ivy D., Alhubaishi A.A., Alkofide H. (2024). Team-Based Learning versus Traditional Teaching Effect on Pharmacy Students’ Performance: A Systematic Review and Meta-Analysis. Saudi Pharm. J..

[15] Burton R., Kellett U., Mansah M., Sriram D. (2024). A Systematic Review of Online Team Based Learning Approaches in Health Professional Education. Nurse Educ. Today.

[16] Vannini V., Alberti S., Epifani C., Valentini O., Ferri P. (2022). The Effects of Online Team-Based Learning on Undergraduate Nursing Students’ Performance, Attitudes and Accountability during COVID-19 Pandemic. Acta Bio Medica Atenei Parm..

[17] Case S.M., Swanson D.B. (1996). Constructing Written Test Questions for the Basic and Clinical Sciences.

[18] Britton E., Simper N., Leger A., Stephenson J. (2017). Assessing Teamwork in Undergraduate Education: A Measurement Tool to Evaluate Individual Teamwork Skills. Assess. Eval. High. Educ..

[19] Valentine M.A., Nembhard I.M., Edmondson A.C. (2015). Measuring Teamwork in Health Care Settings: A Review of Survey Instruments. Med. Care.

[20] Lein D.H., Lowman J.D., Eidson C.A., Yuen H.K. (2017). Cross-Validation of the Student Perceptions of Team-Based Learning Scale in the United States. J. Educ. Eval. Health Prof..

[21] Lucas da Rocha Cunha M., Amendola F., Fernandez Samperiz M.M., Gomes da Costa Mohallem A. (2018). Evaluation of Student Perception of the *Team-Based Learning* Method (APA-TBL): Instrument Construction and Validation. Nurse Educ. Pract..

[22] Mennenga H.A. (2012). Development and Psychometric Testing of the Team-Based Learning Student Assessment Instrument. Nurse Educ..

[23] Ibrahim M.E. (2020). Team-Based Learning Student Assessment Instrument (TBL-SAI) for Assessing Students’ Acceptance of TBL in a Saudi Medical School: Psychometric Analysis and Differences by Academic Year. Saudi Med. J..

[24] Keshmiri F., Rahmati A., Amin A.G., Faezi T. (2016). Validating and Assessing the Reaction of Medical Students Toward Team-Based Learning. Acta Med. Iran..

[25] Souza F., Carvalho E., Castro B., Matos E., Arreguy I. (2019). Team-Based Learning Student Assessment Instrument in Brazilian Dental Education: A Validation Study. J. Dent. Health Oral Disord. Ther..

[26] Nation L.M., Tweddell S., Rutter P. (2016). The Applicability of a Validated Team-Based Learning Student Assessment Instrument to Assess United Kingdom Pharmacy Students’ Attitude toward Team-Based Learning. J. Educ. Eval. Health Prof..

[27] Ulrich D., Brewer T., Steele-Johnson D., Juvina I., Peyton E., Hammond C. (2017). Team-Based Learning’s Effects on Standardized Test Scores and Student Reactions. J. Excell. Coll. Teach..

[28] Elm E.v., Altman D.G., Egger M., Pocock S.J., Gøtzsche P.C., Vandenbroucke J.P. (2008). The Strengthening the Reporting of Observational Studies in Epidemiology (STROBE) Statement: Guidelines for Reporting Observational Studies. J. Clin. Epidemiol..

[29] Alamer A. (2022). Exploratory Structural Equation Modeling (ESEM) and Bifactor ESEM for Construct Validation Purposes: Guidelines and Applied Example. Res. Methods Appl. Linguist..

[30] Marsh H.W., Guo J., Dicke T., Parker P.D., Craven R.G. (2020). Confirmatory Factor Analysis (CFA), Exploratory Structural Equation Modeling (ESEM), and Set-ESEM: Optimal Balance Between Goodness of Fit and Parsimony. Multivar. Behav. Res..

[31] Lubbe D. (2023). Advantages of Using Unweighted Approximation Error Measures for Model Fit Assessment. Psychometrika.

[32] Bagozzi R.P., Yi Y. (1988). On the Evaluation of Structural Equation Models. J. Acad. Mark. Sci..

[33] Hair J.F., Black W.C., Babin B.J., Hair J.F. (2010). Multivariate Data Analysis: A Global Perspective.

[34] Marsh H.W., Morin A.J.S., Parker P.D., Kaur G. (2014). Exploratory Structural Equation Modeling: An Integration of the Best Features of Exploratory and Confirmatory Factor Analysis. Annu. Rev. Clin. Psychol..

[35] Vangone I., Arrigoni C., Magon A., Conte G., Russo S., Belloni S., Stievano A., Alfes C.M., Caruso R. (2024). The Efficacy of High-Fidelity Simulation on Knowledge and Performance in Undergraduate Nursing Students: An Umbrella Review of Systematic Reviews and Meta-Analysis. Nurse Educ. Today.

[36] Andrade H.L., Brookhart S.M. (2020). Classroom Assessment as the Co-Regulation of Learning. Assess. Educ. Princ. Policy Pract..

[37] Prinsen C., Mokkink L., Bouter L., Alonso J., Patrick D., De Vet H., Terwee C. (2018). COSMIN Guideline for Systematic Reviews of Patient-Reported Outcome Measures. Qual. Life Res..

