# Supplementary material for: Translation and Validation of the Italian Version of the Team-Based Learning Student Assessment Instrument (TBL-SAI) in Nursing Students
Source: Nurs Rep. 2025 Jan 17;15(1):26. doi: 10.3390/nursrep15010026 (PMC11768091; doi:10.3390/nursrep15010026)
Supplement: Supplementary file 1 [file nursrep-15-00026-s001.zip › nursrep-3384172-supplementary.pdf]

Supplementary File S1. Power analysis.

```
# Load necessary library
library(lavaan)

# Define the SEM model
model <- '
# Contribution to team (CTT)
CTT =~ ITEM1 + ITEM2 + ITEM3

# Preparation (PREP)
PREP =~ ITEM4 + ITEM5 + ITEM6 + ITEM7 + ITEM8

# Accountability (ACC)
ACC =~ CTT + PREP

# Team-based learning (TBL)
TBL =~ ITEM11 + ITEM13 + ITEM14 + ITEM15 + ITEM17 + ITEM19 + ITEM20 +
ITEM23

# Lecture (LEC)
LEC =~ ITEM9 + ITEM10 + ITEM12 + ITEM16 + ITEM18 + ITEM21 + ITEM22 + ITEM24

# Preference (PREF)
PREF =~ ITEM9 + ITEM10 + ITEM11 + ITEM12 + ITEM13 + ITEM14 + ITEM15 +
ITEM16
PREF =~ ITEM17 + ITEM18 + ITEM19 + ITEM20 + ITEM21 + ITEM22 + ITEM23 +
ITEM24

# Satisfaction (SAT)
SAT =~ ITEM25 + ITEM26 + ITEM27 + ITEM28 + ITEM29 + ITEM30 + ITEM31 +
ITEM32 + ITEM33
```

```
# Covariances
SAT ~~ ACC
SAT ~~ PREF
ACC ~~ TBL
ACC ~~ LEC
TBL ~~ LEC
CTT ~~ PREP
'

# Define the population model
population.model <- '
CTT =~ 0.6*ITEM1 + 0.6*ITEM2 + 0.6*ITEM3
PREP =~ 0.6*ITEM4 + 0.6*ITEM5 + 0.6*ITEM6 + 0.6*ITEM7 + 0.6*ITEM8
ACC =~ 0.6*CTT + 0.6*PREP
TBL =~ 0.6*ITEM11 + 0.6*ITEM13 + 0.6*ITEM14 + 0.6*ITEM15 + 0.6*ITEM17 +
0.6*ITEM19 + 0.6*ITEM20 + 0.6*ITEM23
LEC =~ 0.6*ITEM9 + 0.6*ITEM10 + 0.6*ITEM12 + 0.6*ITEM16 + 0.6*ITEM18 +
0.6*ITEM21 + 0.6*ITEM22 + 0.6*ITEM24
PREF =~ 0.6*ITEM9 + 0.6*ITEM10 + 0.6*ITEM11 + 0.6*ITEM12 + 0.6*ITEM13 +
0.6*ITEM14 + 0.6*ITEM15 + 0.6*ITEM16
PREF =~ 0.6*ITEM17 + 0.6*ITEM18 + 0.6*ITEM19 + 0.6*ITEM20 + 0.6*ITEM21 +
0.6*ITEM22 + 0.6*ITEM23 + 0.6*ITEM24
SAT =~ 0.6*ITEM25 + 0.6*ITEM26 + 0.6*ITEM27 + 0.6*ITEM28 + 0.6*ITEM29 +
0.6*ITEM30 + 0.6*ITEM31 + 0.6*ITEM32 + 0.6*ITEM33
SAT ~~ 0.2*ACC
SAT ~~ 0.2*PREF
ACC ~~ 0.2*TBL
ACC ~~ 0.2*LEC
TBL ~~ 0.2*LEC
CTT ~~ 0.2*PREP
'
```

```
# Set seed for reproducibility
set.seed(12345)
n_sim <- 1000  # Number of simulations
desired_power <- 0.80  # Desired statistical power level
max_iterations <- 20  # Maximum number of iterations
current_iteration <- 0  # Initialize current iteration
sample_size <- 170  # Initial sample size for the simulation

# Function to simulate data and fit the SEM model
simulate_power <- function(n) {
    sim.data <- simulateData(population.model, sample.nobs = n)
    fit <- sem(model, data = sim.data, control = list(tol = 1e-6))
    if (inspect(fit, "converged")) {
        fitMeasures <- fitMeasures(fit, c("pvalue", "rmsea"))
        return(fitMeasures["pvalue"] < 0.05)  # Check if p-value is
significant
    } else {
        return(NA)  # Return NA if model did not converge
    }
}

# Iterative process to find the sample size
estimated_power <- 0
while ((is.na(estimated_power) || estimated_power < desired_power) &&
current_iteration < max_iterations) {
    # Simulate power with current sample size
    powers <- replicate(n_sim, simulate_power(sample_size))
    estimated_power <- mean(powers, na.rm = TRUE)

    # Print the estimated power and current sample size
    cat(sprintf("Iteration %d: Estimated Power = %.2f, Sample Size = %d\n",
current_iteration, estimated_power, sample_size))
```

```
    # Increase sample size if power is insufficient
    sample_size <- sample_size + 20  # Increment sample size
    current_iteration <- current_iteration + 1
}

# Print final sample size estimation
if (estimated_power >= desired_power) {
    cat("Estimated sample size to achieve desired power of 80%:",
sample_size, "\n")
} else {
    cat("Reached maximum number of iterations without achieving desired
power. Estimated sample size:", sample_size, "\n")
}
```
